# Supplementary material for: Development of a CRISPR activation system for targeted gene upregulation in Synechocystis sp. PCC 6803
Source: Commun Biol. 2025 May 21;8:772. doi: 10.1038/s42003-025-08164-y (PMC12095680; doi:10.1038/s42003-025-08164-y)
Supplement: Supplementary file 1 — Supplementary Material [file 42003_2025_8164_MOESM1_ESM.pdf]

SUPPLEMENTARY MATERIAL

Development of a CRISPR activation system for targeted gene upregulation in *Synechocystis* sp. PCC 6803

B. Bourgade, H. Xie, P. Lindblad & K. Stensjö

Contents

Tables 1

Supplementary Table 1. List of *Synechocystis* strains 1

Supplementary Table 2. List of plasmids 2

Supplementary Table 3. List of oligonucleotides 4

Supplementary Table 4. List of relevant DNA sequences 9

Figures 12

Supplementary Figure 1. Schematic representation of CRISPRa plasmids 12

Supplementary Figure 2. Tool stability 13

Supplementary Figure 3. Impact of rhamnose concentration on activation levels 13

Supplementary Figure 4. Growth profiles and IB/3M1B production of CRISPRa-activated strains with multiple *kivD*^{S286T} copies 14

Supplementary Figure 5. Growth profiles of strains with CRISPRa targeting for target mapping 15

Supplementary Figure 6. IB/3M1B ratio for CRISPRa-targeted strains 15

Supplementary Figure 7. Relative transcript levels of target genes 16

Supplementary Figure 8. Simplified metabolic map of IB/3M1B biosynthesis 17

Supplementary Figure 9. Relative transcript levels of multiplexed gene targets 18

Tables

Supplementary Table 1. *Synechocystis* background strains used in this study.

**Strain**	**Genotype**	**Reference**
WT	Wild-type *Synechocystis* sp. PCC 6803	[1]
sBB_CA1	Δ*slr0168*::P_{trc}-*GFP* (Sp^{R}*)	This study
sBB_CA2	Δ*slr0168*::J23119-*GFP* (Sp^{R}*)	This study
sBB_CA3	Δ*slr0168*::J23116-*GFP* (Sp^{R}*)	This study
sBB_CA4	Δ*slr0168*::J23107-*GFP* (Sp^{R}*)	This study
sBB_CA5	Δ*slr0168*::J23101-*GFP* (Sp^{R}*)	This study
ddh_kivD	Δ*ddh*::P_{trc}-$kivd^{S286T}$ (Cm^{R}*)	Obtained with plasmid pHX8[2]
HX11	Δ*ddh*::P_{trc}-$kivd^{S286T}$ (Cm^{R}*), Δ*slr0168*:: P_{trc}-$kivd^{S286T}$ (Sp^{R}*)	[3]
HX51	Δ*ddh*::P_{trc}-$kivd^{S286T}$ (Cm^{R}*); Δ*slr0168*:: P_{trc}-$kivd^{S286T}$ (Sp^{R}*); Δ*sll1564*:: P_{trc}-$kivd^{S286T}$ (Em^{R}*)	Obtained with plasmids pHX8 and pHX15 and a derivative of pHX16[2]

*Sp^{R}: spectinomycin resistance cassette; Cm^{R}: chloramphenicol resistance cassette; Em^{R}: erythromycin resistance cassette

References

1. Williams, J. G. K. Construction of specific mutations in Photosystem II photosynthetic reaction center by genetic engineering methods in *Synechocystis* 6803. *Methods Enzymol.* **167**, 766–778 (1988).

2. Xie, H. & Lindblad, P. Expressing 2-keto acid pathway enzymes significantly increases photosynthetic isobutanol production. *Microb. Cell Fact.* **21**, 1–17 (2022).

3. Xie, H., Bourgade, B., Stensjö, K. & Lindblad, P. dCas12a-mediated CRISPR interference for multiplex gene repression in cyanobacteria for enhanced isobutanol and 3-methyl-1-butanol production. Preprint available at diva2:1895139 (2024).

Supplementary Table 2. List of plasmids used in this study.

Plasmid	Relevant elements	Reference
P3	$HA_{slr0168}$; P_{trc}-*sll1363-slr0452*; SpR*	[1]
pBB_CA1	$HA_{slr0168}$; P_{trc}-GFP; SpR*	This study
pBB_CA2	$HA_{slr0168}$; J23119-GFP; SpR*	This study
pBB_CA3	$HA_{slr0168}$; J23116-GFP; SpR*	This study
pBB_CA4	$HA_{slr0168}$; J23107-GFP; SpR*	This study
pBB_CA5	$HA_{slr0168}$; J23101-GFP; SpR*	This study
pBB_dCas12a	*rhaS*; P_{rha}-dCas12; P_{rha}-$gRNA_{(no\ target)}$; KmR*	[2]
pBB_CA	*rhaS*; P_{rha}-dCas12-$SoxS^{R93A}$; P_{rha}-gRNA(no target); KmR*	This study
pBB_CA6	*rhaS*; P_{rha}-dCas12-$SoxS^{R93A}$; P_{rha}-$gRNA_{(-48)}$; KmR*	This study
pBB_CA7	*rhaS*; P_{rha}-dCas12-$SoxS^{R93A}$; P_{rha}-$gRNA_{(-97\ NTS)}$; KmR*	This study
pBB_CA8	*rhaS*; P_{rha}-dCas12-$SoxS^{R93A}$; P_{rha}-$gRNA_{(-108)}$; KmR*	This study
pBB_CA9	*rhaS*; P_{rha}-dCas12-$SoxS^{R93A}$; P_{rha}-$gRNA_{(-144\ NTS)}$; KmR*	This study
pBB_CA10	*rhaS*; P_{rha}-dCas12-$SoxS^{R93A}$; P_{rha}-$gRNA_{(-156)}$; KmR*	This study
pBB_CA11	*rhaS*; P_{rha}-dCas12-$SoxS^{R93A}$; P_{rha}-$gRNA_{(-251)}$; KmR*	This study
pBB_CA12	*rhaS*; P_{rha}-dCas12-$SoxS^{R93A}$; P_{rha}-$gRNA_{(-328)}$; KmR*	This study
pBB_CA13	*rhaS*; P_{rha}-dCas12-$SoxS^{R93A}$; P_{rha}-$gRNA_{(-108/-156)}$; KmR*	This study
pBB_CA14	*rhaS*; P_{rha}-dCas12-$SoxS^{R93A}$; P_{rha}-$gRNA_{(CDS)}$; KmR*	This study
pBB_CA15	*rhaS*; P_{rha}-dCas12-$SoxS^{R93A}$; P_{rha}-$gRNA_{(J23)}$; KmR*	This study
pBB_CA16	*rhaS*; P_{rha}-dCas12-$SoxS^{R93A}$; P_{rha}-$gRNA_{(ddh)}$; KmR*	This study
pBB_CA17	*rhaS*; P_{rha}-dCas12-$SoxS^{R93A}$; P_{rha}-$gRNA_{(NS1)}$; KmR*	This study
pBB_CA18	*rhaS*; P_{rha}-dCas12-$SoxS^{R93A}$; P_{rha}-$gRNA_{(NS1-ddh)}$; KmR*	This study
pBB_CA19	*rhaS*; P_{rha}-dCas12-$SoxS^{R93A}$; P_{rha}-$gRNA_{(sll1654)}$; KmR*	This study
pBB_CA20	*rhaS*; P_{rha}-dCas12-$SoxS^{R93A}$; P_{rha}-$gRNA_{(NS1-sll1564)}$; KmR*	This study
pBB_CA21	*rhaS*; P_{rha}-dCas12-$SoxS^{R93A}$; P_{rha}-$gRNA_{(ddh-sll1564)}$; KmR*	This study
pBB_CA22	*rhaS*; P_{rha}-dCas12-$SoxS^{R93A}$; P_{rha}-$gRNA_{(Triple)}$; KmR*	This study
pBB_CA23	*rhaS*; P_{rha}-dCas12-$SoxS^{R93A}$; P_{rha}-$gRNA_{(pyk2.1)}$; KmR*	This study
pBB_CA24	*rhaS*; P_{rha}-dCas12-$SoxS^{R93A}$; P_{rha}-$gRNA_{(pyk2.2)}$; KmR*	This study
pBB_CA25	*rhaS*; P_{rha}-dCas12-$SoxS^{R93A}$; P_{rha}-$gRNA_{(pyk1.1)}$; KmR*	This study

pBB_CA26	*rhaS*; P_{rha}-dCas12-SoxS^{R93A}; P_{rha}-gRNA$_{(pyk1.2)}$; KmR*	This study
pBB_CA27	*rhaS*; P_{rha}-dCas12-SoxS^{R93A}; P_{rha}-gRNA$_{(pntA.1)}$; KmR*	This study
pBB_CA28	*rhaS*; P_{rha}-dCas12-SoxS^{R93A}; P_{rha}-gRNA$_{(pntA.2)}$; KmR*	This study
pBB_CA29	*rhaS*; P_{rha}-dCas12-SoxS^{R93A}; P_{rha}-gRNA$_{(ME)}$; KmR*	This study
pBB_CA30	*rhaS*; P_{rha}-dCas12-SoxS^{R93A}; P_{rha}-gRNA$_{(tpi)}$; KmR*	This study
pBB_CA31	*rhaS*; P_{rha}-dCas12-SoxS^{R93A}; P_{rha}-gRNA$_{(petH)}$; KmR*	This study
pBB_CA32	*rhaS*; P_{rha}-dCas12-SoxS^{R93A}; P_{rha}-gRNA$_{(acnSP)}$; KmR*	This study
pBB_CA33	*rhaS*; P_{rha}-dCas12-SoxS^{R93A}; P_{rha}-gRNA$_{(slr6040)}$; KmR*	This study
pBB_CA34	*rhaS*; P_{rha}-dCas12-SoxS^{R93A}; P_{rha}-gRNA$_{(pyk2.1\text{- }pyk2.2)}$; KmR*	This study
pBB_CA35	*rhaS*; P_{rha}-dCas12-SoxS^{R93A}; P_{rha}-gRNA$_{(pyk1.1\text{- }pyk1.2)}$; KmR*	This study
pBB_CA36	*rhaS*; P_{rha}-dCas12-SoxS^{R93A}; P_{rha}-gRNA$_{(pyk2.1\text{- }pyk1.1)}$; KmR*	This study
pBB_CA37	*rhaS*; P_{rha}-dCas12-SoxS^{R93A}; P_{rha}-gRNA$_{(pyk2.1\text{-ME})}$; KmR*	This study
pBB_CA38	*rhaS*; P_{rha}-dCas12-SoxS^{R93A}; P_{rha}-gRNA$_{(acnSP\text{-ME})}$; KmR*	This study
pBB_CA39	*rhaS*; P_{rha}-dCas12-SoxS^{R93A}; P_{rha}-gRNA$_{(pyk2.1\text{- }slr6040)}$; KmR*	This study
pBB_CA40	*rhaS*; P_{rha}-dCas12-SoxS^{R93A}; P_{rha}-gRNA$_{(slr6040\text{-ME})}$; KmR*	This study

*Sp^R: spectinomycin resistance cassette; Km^R: kanamycin resistance cassette

References

1. Xie, H., Kjellström, J. & Lindblad, P. Sustainable production of photosynthetic isobutanol and 3-methyl-1-butanol in the cyanobacterium *Synechocystis* sp. PCC 6803. *Biotechnol. Biofuels Bioprod.* **16**, 1–17 (2023).

2. Xie, H., Bourgade, B., Stensjö, K. & Lindblad, P. dCas12a-mediated CRISPR interference for multiplex gene repression in cyanobacteria for enhanced isobutanol and 3-methyl-1-butanol production. Preprint available at diva2:1895139 (2024).

Supplementary Table 3. Oligonucleotides used in this study.

**I. Primers for building plasmids**	
Backbone_Fwd	AGATCTAACTACCGCATTAAAG
Backbone_Rev	GATTATCGGCACCGTCTCTAATTTTAACGTGGCTTTGCGC
dCas12_Fwd	TTAATGCGGTAGTTAGATCTTTGACAGCTAGCTCAGTCC
dCas12_Rev	AGCGCGTCCAGATCCAGAAGCCTCAGATCCGTTATTCCTATTCTGCACGA
SoxS_Fwd	GGATCTGAGGCTTCTGGATCTGGACGCGCTTCCCACCAGAAAATCATCCA
SoxS_Rev	TTAGAGACGGTGCCGATAATCTTAGAGACGGTGCCGATAATC
Ptrc_Fwd	ATATATGAATTCGAGCTGTTGACAATTGTGAG
Ptrc_Rev	GAAAAGTTCTTCTCCTTTACTCATCATTAGAAAACCTCCTTAGC
GFP_Fwd	TCATGCTAAGGAGGTTTTCTAATGATGAGTAAAGGAGAAGAACT
GFP_Rev	ATATATGCGGCCGCTTATTTGTATAGTTCATCCA
J23119_Fwd	ATATATGAATTCTTGACAGCTAGCTCAGTCCTAGGTATAATGCTAGCGGGCCCAAGTTCACTT
J23116_Fwd	ATATATGAATTCTTGACAGCTAGCTCAGTCCTAGGGACTATGCTAGCTGGGCCCAAGTTCACTT
J23107_Fwd	ATATATGAATTCTTTACGGCTAGCTCAGCCCTAGGTATTATGCTAGCTGGGCCCAAGTTCACTT
J23101_Fwd	ATATATGAATTCTTTACAGCTAGCTCAGTCCTAGGTATTATGCTAGCTGGGCCCAAGTTCACTT
**II. Primers for cloning gRNAs**	
-48_Fwd	5’Phos/ AGATAATTCGAGCTGTTGACAATT
-48_Rev	5’Phos/ AGACAATTGTCAACAGCTCGAATT
-97_Fwd	5’Phos/ AGATATTTTAGATTAATTCAACAG
-97_Rev	5’Phos/ AGACCTGTTGAATTAATCTAAAAT
-108_Fwd	5’Phos/ AGATTGAAATATTACTGTTGAATT
-108_Rev	5’Phos/ AGACAATTCAACAGTAATATTTCA
-144_Fwd	5’Phos/ AGATAACTCGCAATAATTGCATTA
-144_Rev	5’Phos/ AGACTAATGCAATTATTGCGAGTT
-156_Fwd	5’Phos/ AGATAATCAACTTAATTAATGCAA
-156_Rev	5’Phos/ AGACTTGCATTAATTAAGTTGATT
-251_Fwd	5’Phos/ AGATATTGAAGAAATGGCCCTGGA
-251_Rev	5’Phos/ AGACTCCAGGGCCATTTCTTCAAT
-328_Fwd	5’Phos/ AGATTTCATTGTGTTAGGGGAGGT

-328_Rev	5'Phos/ AGACACCTCCCCTAACACAATGAA
CDS_Fwd	5'Phos/ AGATTCTTATGGTGTTCAATGCTT
CDS_Rev	5'Phos/ AGACAAGCATTGAACACCATAAGA
-108/-156_Fwd	5'Phos/ AGATAATCAACTTAATTAATGCAAGTCTAAGAACTTTAAATAATTTCTACTGTTGTAGATTGAAATATTACTGTTGAATT
-108/-156_Rev	5'Phos/ AGACAATTCAACAGTAATATTTCAATCTACAACAGTAGAAATTATTTAAAGTTCTTAGACTTGCATTAATTAAGTTGATT
ddh_Fwd	5'Phos/ AGATCAAACACGTTCTAAACTACT
ddh_Rev	5'Phos/ AGACAGTAGTTTAGAACGTGTTTG
NS1_Fwd	5'Phos/ AGATAATCAACTTAATTAATGCAA
NS1_Rev	5'Phos/ AGACTTGCATTAATTAAGTTGATT
sll1564_Fwd	5'Phos/ AGATAAATCCAGTAACTACATAAT
sll1564_Rev	5'Phos/ AGACATTATGTAGTTACTGGATTT
NS1-ddh_Fwd	5'Phos/ AGATCAAACACGTTCTAAACTACTGTCTAAGAACTTTAAATAATTTCTACTGTTGTAGATAATCAACTTAATTAATGCAA
NS1-ddh_Rev	5'Phos/ AGACTTGCATTAATTAAGTTGATTATCTACAACAGTAGAAATTATTTAAAGTTCTTAGACAGTAGTTTAGAACGTGTTTG
NS1-sll1564_Fwd	5'Phos/ AGATAATCAACTTAATTAATGCAAGTCTAAGAACTTTAAATAATTTCTACTGTTGTAGATAAATCCAGTAACTACATAAT
NS1-sll1564_Rev	5'Phos/ AGACATTATGTAGTTACTGGATTTATCTACAACAGTAGAAATTATTTAAAGTTCTTAGACTTGCATTAATTAAGTTGATT
ddh-sll1564_Fwd	5'Phos/ AGATCAAACACGTTCTAAACTACTGTCTAAGAACTTTAAATAATTTCTACTGTTGTAGATAAATCCAGTAACTACATAAT
ddh-sll1564_Rev	5'Phos/ AGACATTATGTAGTTACTGGATTTATCTACAACAGTAGAAATTATTTAAAGTTCTTAGACAGTAGTTTAGAACGTGTTTG
Triple1_Fwd	5'Phos/ AGATCAAACACGTTCTAAACTACTGTCTAAGAA

	CTTTAAATAATTTCTACTGTTGTAGATAATCAAC TTAATTAATGCAA
Triple1_Rev	ATCTACAACAGTAGAAATTATTTAAAGTTCTTAG ACAGTAGTTTAGAACGTGTTTG
Triple2_Fwd	GTCTAAGAACTTTAAATAATTTCTACTGTTGTAG ATAAATCCAGTAACTACATAAT
Triple2_Rev	5’Phos/ AGACATTATGTAGTTACTGGATTTATCTACAAC AGTAGAAATTATTTAAAGTTCTTAGACTTGCATT AATTAAGTTGATT
pyk2.1_Fwd	5’Phos/ AGATAGTTGCATCGGCTTACAGGG
pyk2.1_Rev	5’Phos/ AGACCCCTGTAAGCCGATGCAACT
pyk2.2_Fwd	5’Phos/ AGATGGCAATTTTTCCCAATAGTC
pyk2.2_Rev	5’Phos/ AGACGACTATTGGGAAAAATTGCC
pyk1.1_Fwd	5’Phos/ AGATAAAATTAAAACCGCCGGTAA
pyk1.1_Rev	5’Phos/ AGACTTACCGGCGGTTTTAATTTT
pyk1.2_Fwd	5’Phos/ AGATCCCGGCAAAATATAATCCAG
pyk1.2_Rev	5’Phos/ AGACCTGGATTATATTTTGCCGGG
pntA.1_Fwd	5’Phos/ AGATCGCTGTCCCCGTAAGATGGG
pntA.1_Rev	5’Phos/ AGACCCCATCTTACGGGGACAGCG
pntA.2_Fwd	5’Phos/ AGATCTCACAAATTAGCCTTAACG
pntA.2_Rev	5’Phos/ AGACCGTTAAGGCTAATTTGTGAG
ME_Fwd	5’Phos/ AGATCCCATGTATCTAGCCCCATC
ME_Rev	5’Phos/ AGACGATGGGGCTAGATACATGGG
Tpi_Fwd	5’Phos/ AGATAATCTAGTCCAATCGAGAAG
Tpi_Rev	5’Phos/ AGACCTTCTCGATTGGACTAGATT
petH_Fwd	5’Phos/ AGATCCGGACGATTAACCCTGGAA
petH_Rev	5’Phos/ AGACTTCCAGGGTTAATCGTCCGG
pyk2.1.2_Fwd	5’Phos/ AGATAGTTGCATCGGCTTACAGGGGTCTAAGA ACTTTAAATAATTTCTACTGTTGTAGATGGCAAT TTTTCCCAATAGTC
pyk2.1.2_Rev	5’Phos/ AGACGACTATTGGGAAAAATTGCCATCTACAAC AGTAGAAATTATTTAAAGTTCTTAGACCCCTGT AAGCCGATGCAACT
pyk1.1.2_Fwd	5’Phos/ AGATAAAATTAAAACCGCCGGTAAGTCTAAGAA

	CTTTAAATAATTTCTACTGTTGTAGATCCCGGC AAAATATAATCCAG
pyk1.1.2_Rev	5’Phos/ AGACCTGGATTATATTTTGCCGGGATCTACAAC AGTAGAAATTATTTAAAGTTCTTAGACTTACCG GCGGTTTTAATTTT
pyk2.1-pyk1.1_Fwd	5’Phos/ AGATAGTTGCATCGGCTTACAGGGGTCTAAGA ACTTTAAATAATTTCTACTGTTGTAGATAAAATT AAAACCGCCGGTAA
pyk2.1-pyk1.1_Rev	5’Phos/ AGACTTACCGGCGGTTTTAATTTTATCTACAAC AGTAGAAATTATTTAAAGTTCTTAGACCCCTGT AAGCCGATGCAACT
pyk2.1-ME_Fwd	5’Phos/ AGATAGTTGCATCGGCTTACAGGGGTCTAAGA ACTTTAAATAATTTCTACTGTTGTAGATCCCATG TATCTAGCCCCATC
pyk2.1-ME_Rev	5’Phos/ AGACGATGGGGCTAGATACATGGGATCTACAA CAGTAGAAATTATTTAAAGTTCTTAGACCCCTG TAAGCCGATGCAACT
slr6040_Fwd	5’Phos/ AGATGACCAGGGTTGGGTGAACTA
slr6040_Rev	5’Phos/ AGACTAGTTCACCCAACCCTGGTC
slr6040-ME_Fwd	5’Phos/ AGATGACCAGGGTTGGGTGAACTAGTCTAAGA ACTTTAAATAATTTCTACTGTTGTAGATCCCATG TATCTAGCCCCATC
slr6040-ME_Rev	5’Phos/ AGACGATGGGGCTAGATACATGGGATCTACAA CAGTAGAAATTATTTAAAGTTCTTAGACTAGTTC ACCCAACCCTGGTC
slr6040-pyk2.1_Fwd	5’Phos/ AGATGACCAGGGTTGGGTGAACTAGTCTAAGA ACTTTAAATAATTTCTACTGTTGTAGATAGTTGC ATCGGCTTACAGGG
slr6040- pyk2.1_Rev	5’Phos/ AGACCCCTGTAAGCCGATGCAACTATCTACAA CAGTAGAAATTATTTAAAGTTCTTAGACTAGTTC ACCCAACCCTGGTC
acnSP_Fwd	5’Phos/ AGATGCGATTGGCAGCGTGGCGAC
acnSP_Rev	5’Phos/ AGACGTCGCCACGCTGCCAATCGC
acnSP-ME_Fwd	AGATGCGATTGGCAGCGTGGCGACGTCTAAGA ACTTTAAATAATTTCTACTGTTGTAGATCCCATG TATCTAGCCCCATC
acnSP-ME_Rev	AGACGATGGGGCTAGATACATGGGATCTACAA CAGTAGAAATTATTTAAAGTTCTTAGACGTCGC CACGCTGCCAATCGC

**III. Primers for RT-qPCR**	
q.rnpB_Fwd	CGTTAGGATAGTGCCACAG
q.rnpB_Rev	CGCTCTTACCGCACCTTTG
q.GFP_Fwd	GATGGAAGCGTTCAACTAGCA
q.GFP_Rev	GCAGATTGTGTGGACAGGTAAT
q.KivD_Fwd	GAGTGAACCCAACCTGAAAGA
q.KivD_Rev	TGGGTAAAGGCTCCAGTAGA
q.Flag_Fwd	GACTACAAGGATGACGATGACAA
q.His_Fwd	CATCACCATCACCACGGTAG
q.Tag_Rev	GTTCATGTAAGCGGTCCAGTAA
q.pyk1_Fwd	TGGAAGCGAGGAGAGAAGTA
q.pyk1_Rev	AACCCATGCCCTAAGTGAAG
q.pyk2_Fwd	TCCAGCCCGAACATCAAATC
q.pyk2_Rev	GGGAACCCTTTCCAGCAATAA
q.pntA_Fwd	CCAGTCAATCCAGTCAGCTTTA
q.pntA_Rev	CCTCATCTTCCACATCCACTTT
q.ME_Fwd	GGCAGTGAAAGCTCTGGATAA
q.ME_Rev	AATGCGACTAACCACGCTAAT
q.tpi_Fwd	CAATCTAACCTAGTCATCGCCTAC
q.tpi_Rev	TCCCGAATCAGCCCAATAAC
q.petH_Fwd	CCGTTACCTAGAAGGGCAAAG
q.petH_Rev	GTCTGGTGGAAGCAATGGAATA
q.slr6040_Fwd	GATCTAGGAATGGTGCTGGAAA
q.slr6040_Rev	CACCCAACCCTGGTCTAAAT
q.acnSP_Fwd	CGTCGCCACGCTGCCAATCG
q.acnSP_Rev	GGATTTTTAGCAGTTCACAT

Supplementary Table 4. DNA sequences of relevant CRISPRa elements used in this study.

**Element**	**DNA sequence**	**Reference**
rhaS	ATGACCGTATTACATAGTGTGGATTTTTTTCCGTCTGGTA<br>ACGCGTCCGTGGCGATAGAACCCCGGCTCCCGCAGGC<br>GGATTTTCCTGAACATCATCATGATTTTCATGAAATTGTG<br>ATTGTCGAACATGGCACGGGTATTCATGTGTTTAATGGG<br>CAGCCCTATACCATCACCGGTGGCACGGTCTGTTTCGTA<br>CGCGATCATGATCGGCATCTGTATGAACATACCGATAAT<br>CTGTGTCTGACCAATGTGCTGTATCGCTCGCCGGATCGA<br>TTTCAGTTTCTCGCCGGGCTGAATCAGTTGCTGCCACAA<br>GAGCTGGATGGGCAGTATCCGTCTCACTGGCGCGTTAA<br>CCACAGCGTCTTGCAGCAAGTGCGACAGCTGGTTGCAC<br>AGATGGAACAGCAGGAAGGGGAAAATGATTTACCCTCGA<br>CCGCCAGTCGCGAGATCTTGTTTATGCAATTACTGCTCT<br>TGCTGCGTAAAAGCAGTTTGCAGGAGAACCTGGAAAACA<br>GCGCATCACGTCTCAACTTGCTTCTGGCCTGGCTGGAG<br>GACCATTTTGCCGATGAGGTGAATTGGGATGCCGTGGC<br>GGATCAATTTTCTCTTTCACTGCGTACGCTACATCGGCA<br>GCTTAAGCAGCAAACGGGACTGACGCCTCAGCTATACCT<br>GAACCGCCTGCGATTGATGAAAGCCCGACATCTGCTAC<br>GCCACAGCGAGGCCAGCGTTACTGACATCGCCTATCGC<br>TGTGGATTCAGCGACAGTAACCACTTTTCGACGCTTTTTC<br>GCCGAGAGTTTAACTGGTCACCGCGTGATATTCGCCAG<br>GGACGGGATGGCTTTCTGCAATAA	1
P_{rha}	GCCACAATTCAGCAAATTGTGAACATCATCACGTTCATCT<br>TTCCCTGGTTGCCAATGGCCCATTTTCCTGTCAGTAACG<br>AGAAGGTCGCGAATTCAGGCGCTTTTTAGACTGGTCGTA<br>ATGAA	2
dCas12a	ATGTCAATTTATCAAGAATTTGTTAATAAATATAGTTTAAG<br>TAAAACTCTAAGATTTGAGTTAATCCCACAGGGTAAAACA<br>CTTGAAAACATAAAAGCAAGAGGTTTGATTTTAGATGATG<br>AGAAAAGAGCTAAAGACTACAAAAAGGCTAAACAAATAAT<br>TGATAAATATCATCAGTTTTTTATAGAGGAGATATTAAGTT<br>CGGTTTGTATTAGCGAAGATTTATTACAAAACTATTCTGA<br>TGTTTATTTTAAACTTAAAAAGAGTGATGATGATAATCTAC<br>AAAAAGATTTTAAAAGTGCAAAAGATACGATAAAGAAACA<br>AATATCTGAATATATAAAGGACTCAGAGAAATTTAAGAAT<br>TTGTTTAATCAAAACCTTATCGATGCTAAAAAAGGGCAAG<br>AGTCAGATTTAATTCTATGGCTAAAGCAATCTAAGGATAA<br>TGGTATAGAACTATTTAAAGCCAATAGTGATATCACAGAT<br>ATAGATGAGGCGTTAGAAATAATCAAATCTTTTAAAGGTT<br>GGACAACTTATTTTAAGGGTTTTCATGAAAATAGAAAAAA<br>TGTTTATAGTAGCAATGATATTCCTACATCTATTATTTATA<br>GGATAGTAGATGATAATTTGCCTAAATTTCTAGAAAATAA<br>AGCTAAGTATGAGAGTTTAAAAGACAAAGCTCCAGAAGC<br>TATAAACTATGAACAAATTAAAAAAGATTTGGCAGAAGAG<br>CTAACCTTTGATATTGACTACAAAACATCTGAAGTTAATC<br>AAAGAGTTTTTTCACTTGATGAAGTTTTTGAGATAGCAAA<br>CTTTAATAATTATCTAAATCAAAGTGGTATTACTAAATTTA	3

	ATACTATTATTGGTGGTAAATTTGTAAATGGTGAAAATAC<br>AAAGAGAAAAGGTATAAATGAATATATAAATCTATACTCA<br>CAGCAAATAAATGATAAAACACTCAAAAAATATAAAATGA<br>GTGTTTTATTTAAGCAAATTTTAAGTGATACAGAATCTAAA<br>TCTTTTGTAATTGATAAGTTAGAAGATGATAGTGATGTAG<br>TTACAACGATGCAAAGTTTTTATGAGCAAATAGCAGCTTT<br>TAAAACAGTAGAAGAAAAATCTATTAAAGAAACACTATCT<br>TTATTATTTGATGATTTAAAAGCTCAAAAACTTGATTTGAG<br>TAAAATTTATTTTAAAAATGATAAATCTCTTACTGATCTAT<br>CACAACAAGTTTTTGATGATTATAGTGTTATTGGTACAGC<br>GGTACTAGAATATATAACTCAACAAATAGCACCTAAAAAT<br>CTTGATAACCCTAGTAAGAAAGAGCAAGAATTAATAGCC<br>AAAAAAACTGAAAAAGCAAAATACTTATCTCTAGAAACTA<br>TAAAGCTTGCCTTAGAAGAATTTAATAAGCATAGAGATAT<br>AGATAAACAGTGTAGGTTTGAAGAAATACTTGCAAACTTT<br>GCGGCTATTCCGATGATATTTGATGAAATAGCTCAAAACA<br>AAGACAATTTGGCACAGATATCTATCAAATATCAAAATCA<br>AGGTAAAAAAGACCTACTTCAAGCTAGTGCGGAAGATGA<br>TGTTAAAGCTATCAAGGATCTTTTAGATCAAACTAATAAT<br>CTCTTACATAAACTAAAAATATTTCATATTAGTCAGTCAGA<br>AGATAAGGCAAATATTTTAGACAAGGATGAGCATTTTTAT<br>CTAGTATTTGAGGAGTGCTACTTTGAGCTAGCGAATATA<br>GTGCCTCTTTATAACAAAATTAGAAACTATATAACTCAAA<br>AGCCATATAGTGATGAGAAATTTAAGCTCAATTTTGAGAA<br>CTCGACTTTGGCTAATGGTTGGGATAAAAATAAAGAGCC<br>TGACAATACGGCAATTTTATTTATCAAAGATGATAAATATT<br>ATCTGGGTGTGATGAATAAGAAAAATAACAAAATATTTGA<br>TGATAAAGCTATCAAAGAAAATAAAGGCGAGGGTTATAA<br>AAAAATTGTTTATAAACTTTTACCTGGCGCAAATAAAATG<br>TTACCTAAGGTTTTCTTTTCTGCTAAATCTATAAAATTTTA<br>TAATCCTAGTGAAGATATACTTAGAATAAGAAATCATTCC<br>ACACATACAAAAAATGGTAGTCCTCAAAAAGGATATGAAA<br>AATTTGAGTTTAATATTGAAGATTGCCGAAAATTTATAGAT<br>TTTTATAAACAGTCTATAAGTAAGCATCCGGAGTGGAAAG<br>ATTTTGGATTTAGATTTTCTGATACTCAAAGATATAATTCT<br>ATAGATGAATTTTATAGAGAAGTTGAAAATCAAGGCTACA<br>AACTAACTTTTGAAAATATATCAGAGAGCTATATTGATAG<br>CGTAGTTAATCAGGGTAAATTGTACCTATTCCAAATCTAT<br>AATAAAGATTTTTCAGCTTATAGCAAAGGGCGACCAAATC<br>TACATACTTTATATTGGAAAGCGCTGTTTGATGAGAGAAA<br>TCTTCAAGATGTGGTTTATAAGCTAAATGGTGAGGCAGA<br>GCTTTTTTATCGTAAACAATCAATACCTAAAAAAATCACTC<br>ACCCAGCTAAAGAGGCAATAGCTAATAAAAACAAAGATA<br>ATCCTAAAAAAGAGAGTGTTTTTGAATATGATTTAATCAA<br>AGATAAACGCTTTACTGAAGATAAGTTTTTCTTTCACTGT<br>CCTATTACAATCAATTTTAAATCTAGTGGAGCTAATAAGT<br>TTAATGATGAAATCAATTTATTGCTAAAAGAAAAAGCAAA<br>TGATGTTCATATATTAAGTATAGCTAGAGGTGAAAGACAT<br>TTAGCTTACTATACTTTGGTAGATGGTAAAGGCAATATCA<br>TCAAACAAGATACTTTCAACATCATTGGTAATGATAGAAT<br>GAAAACAAACTACCATGATAAGCTTGCTGCAATAGAGAA	

	AGATAGGGATTCAGCTAGGAAAGACTGGAAAAAGATAAA TAACATCAAAGAGATGAAAGAGGGCTATCTATCTCAGGT AGTTCATGAAATAGCTAAGCTAGTTATAGAGTATAATGCT ATTGTGGTTTTTGAGGATTTAAATTTTGGATTTAAAAGAG GGCGTTTCAAGGTAGAGAAGCAGGTCTATCAAAAGTTAG AAAAAATGCTAATTGAGAAACTAAACTATCTAGTTTTCAA AGATAATGAGTTTGATAAAACTGGGGGAGTGCTTAGAGC TTATCAGCTAACAGCACCTTTTGAGACTTTTAAAAAGATG GGTAAACAAACAGGTATTATCTACTATGTACCAGCTGGTT TTACTTCAAAAATTTGTCCTGTAACTGGTTTTGTAAATCA GTTATATCCTAAGTATGAAAGTGTCAGCAAATCTCAAGAG TTCTTTAGTAAGTTTGACAAGATTTGTTATAACCTTGATAA GGGCTATTTTGAGTTTAGTTTTGATTATAAAAACTTTGGT GACAAGGCTGCCAAAGGCAAGTGGACTATAGCTAGCTTT GGGAGTAGATTGATTAACTTTAGAAATTCAGATAAAAATC ATAATTGGGATACTCGAGAAGTTTATCCAACTAAAGAGTT GGAGAAATTGCTAAAAGATTATTCTATCGAATATGGGCAT GGCGAATGTATCAAAGCAGCTATTTGCGGTGAGAGCGA CAAAAAGTTTTTTGCTAAGCTAACTAGTGTCCTAAATACT ATCTTACAAATGCGTAACTCAAAAACAGGTACTGAGTTAG ATTATCTAATTTCACCAGTAGCAGATGTAAATGGCAATTT CTTTGATTCGCGACAGGCGCCAAAAAATATGCCTCAAGA TGCTGATGCCAATGGTGCTTATCATATTGGGCTAAAAGG TCTGATGCTACTAGGTAGGATCAAAAATAATCAAGAGGG CAAAAAACTCAATTTGGTTATCAAAAATGAAGAGTATTTT GAGTTCGTGCAGAATAGGAATAAC	
SoxS^{R93A}	TCCCACCAGAAAATCATCCAAGATCTGATTGCATGGATC GACGAGCACATTGACCAGCCTCTCAATATCGATGTAGTT GCAAAGAAGAGCGGATACAGCAAATGGTATCTGCAACG CATGTTCCGCACTGTTACACACCAAACATTGGGAGATTA TATTCGCCAACGACGACTCCTGCTGGCAGCCGTAGAGTT ACGAACTACAGAGCGTCCTATTTTTGACATTGCTATGGAT TTGGGTTATGTGAGCCAGCAGACATTCTCCCGTGTGTTT GCGCGTCAGTTTGACCGTACTCCCTCCGATTATCGGCAC CGTCTCTAA	This study

References

1. Xie, H., Bourgade, B., Stensjö, K. & Lindblad, P. dCas12a-mediated CRISPR interference for multiplex gene repression in cyanobacteria for enhanced isobutanol and 3-methyl-1-butanol production. Preprint available at diva2:1895139 (2024).

2. Behle, A., Saake, P., Germann, A. T., Dienst, D. & Axmann, I. M. Comparative dose-response analysis of inducible promoters in cyanobacteria. *ACS Synth. Biol.* **9**, 843–855 (2020).

3. Knoot, C. J., Biswas, S. & Pakrasi, H. B. Tunable repression of key photosynthetic processes using Cas12a CRISPR interference in the fast-growing cyanobacterium *Synechococcus* sp. UTEX 2973. *ACS Synth. Biol.* **9**, 132–143 (2020).

Figures

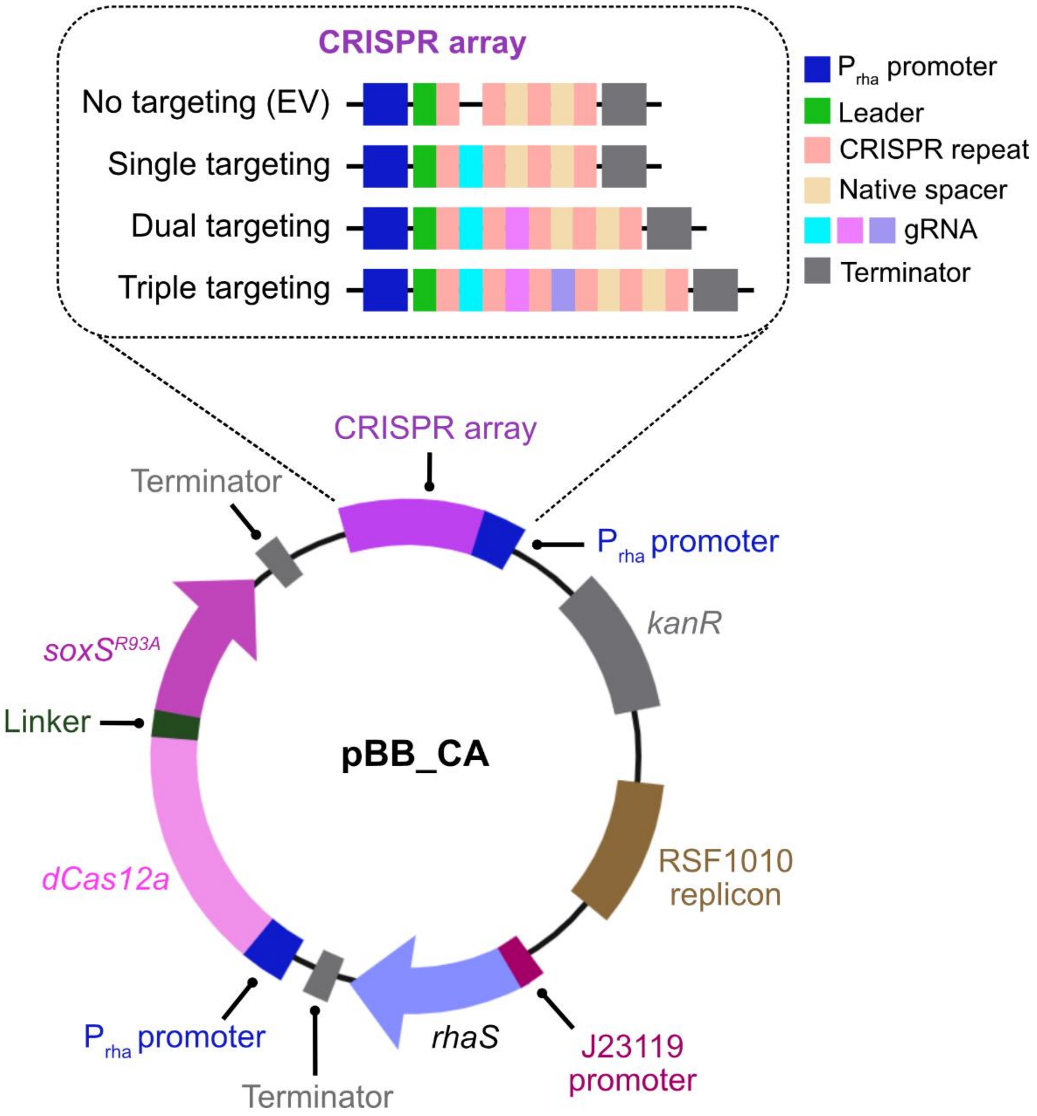

Supplementary Figure 1. Schematic representation of the CRISPRa plasmids (pBB_CA and pBB_CA6-40) used in this study to express the dCas12a-SoxS fusion and gRNA(s). CRISPR components were driven by the rhamnose-inducible P_{rha} promoter, regulated by the RhaS under the constitute J23119 promoter. The CRISPR arrays were designed based on the structure previously described by Ungerer and Pakrasi (2012), incorporating the native leader, repeat and terminator sequences, along with the first two spacers from *Francisella novicida* CRISPR array. Single, dual or triple gRNAs were inserted into the array as detailed in the Methods section. The negative control (EV) contained all CRISPR elements but lacked a targeting gRNA. kanR: kanamycin resistance gene; RSF1010 replicon: broad host-range replicon for plasmid maintenance in *Synechocystis*.

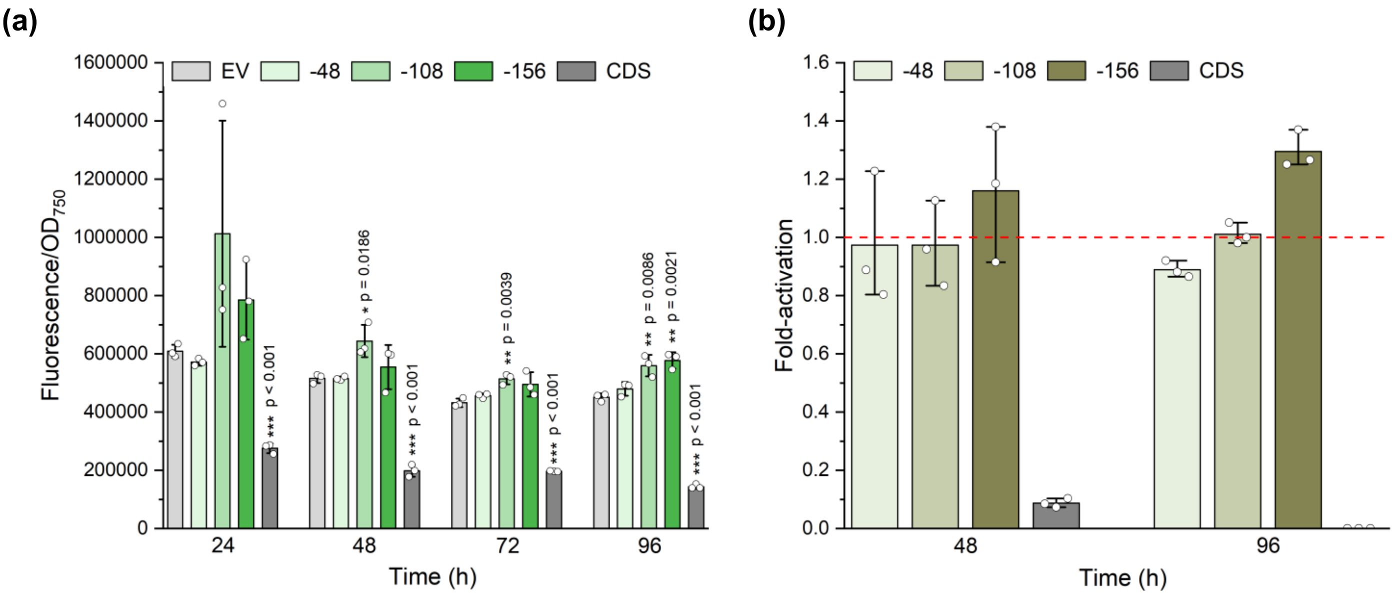

Supplementary Figure 2. GFP activation throughout a 96-hour post-induction period with four selected gRNAs. (a) GFP fluorescence was quantified at 24, 48, 72 and 96 hours after rhamnose induction for four selected gRNAs. (b) Fold-activation was calculated relative to the negative control (EV) at 48h and 96h. EV: negative control – sBB_CA1 expressing plasmid pBB_CA; CDS: coding sequence. Error bars indicate standard deviation (n=3). p value representation: * < 0.05; ** < 0.01; *** < 0.001. p value was calculated by comparing each sample to the respective negative control.

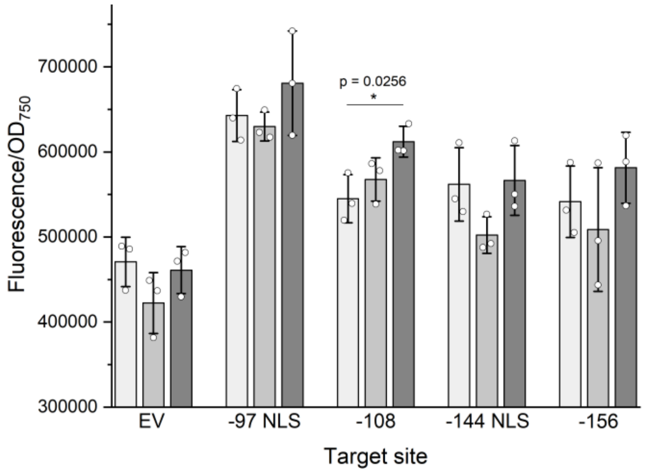

Supplementary Figure 3. Correlation between rhamnose concentration and GFP fluorescence 72 hours post-induction for four selected gRNAs. GFP fluorescence was measured 72 hours post-induction with 3; 6 or 9 mM of rhamnose for four selected gRNAs. EV: negatve control – sBB_CA1 expressing plasmid pBB_CA. Error bars indicate standard deviation (n=3). p value representation: * < 0.05. p value was calculated by comparing each sample to the respective negative control.

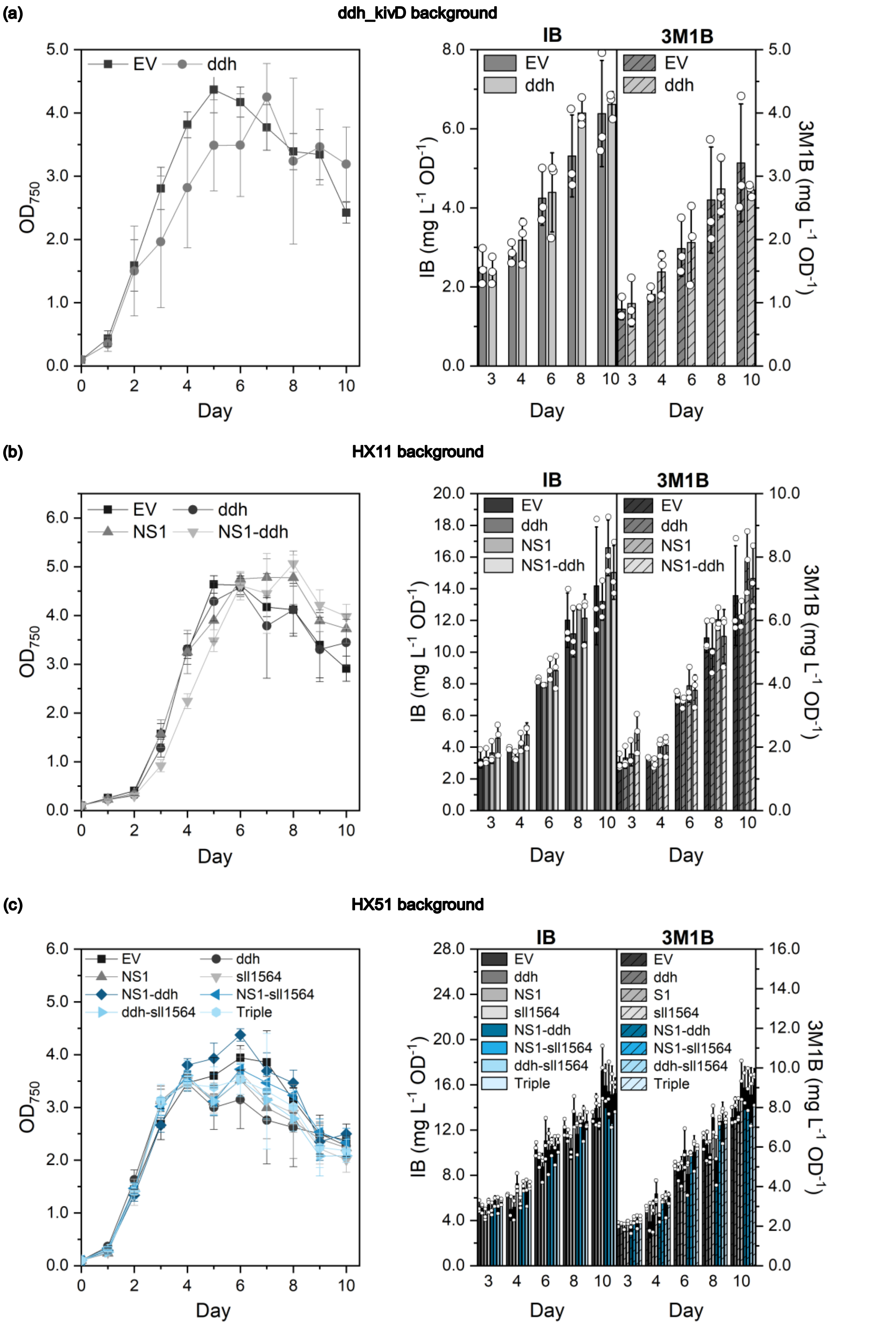

Supplementary Figure 4. Growth profiles and isobutanol (IB)/3-methyl-1-butanol (3M1B) production of CRISPR-activated strains. Growth and corresponding IB/3M1B titres were monitored in CRISPRa-activated derivatives of (a) ddh_kivD, (b) HX11 and (c) HX51 background strains during 10 days post-induction with 3 mM rhamnose**.** EV: negative control – respective background strain expressing plasmid pBB_CA. Error bars represent standard deviation (n=3).

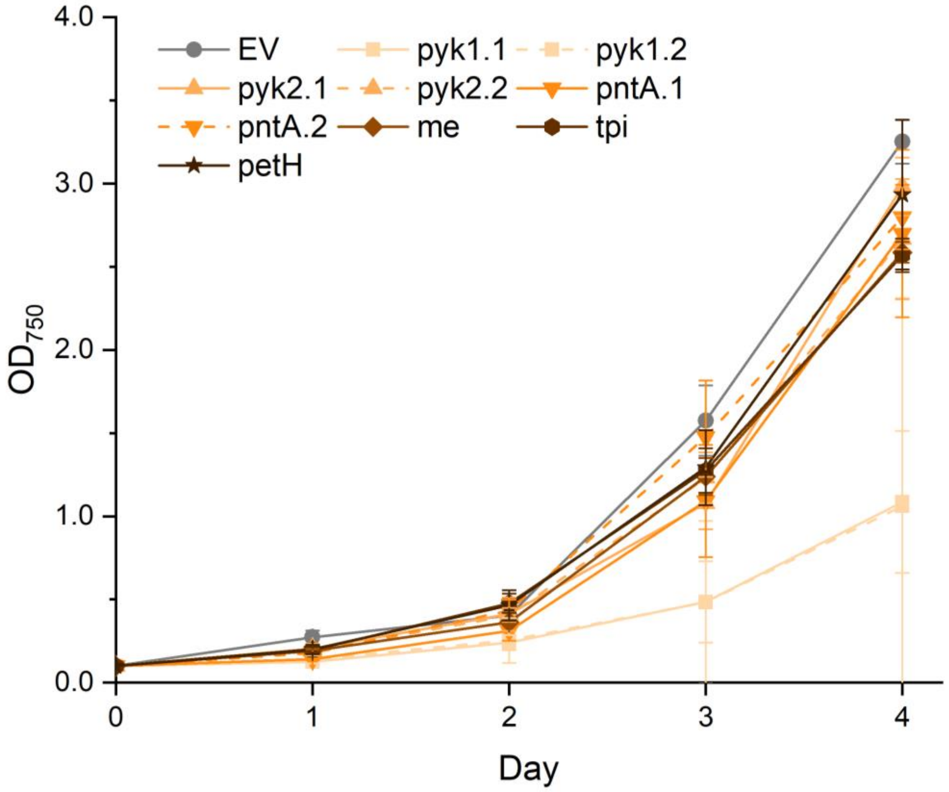

Supplementary Figure 5. Growth profiles of strains with CRISPRa targeting for target mapping. EV: negative control – HX11 expressing plasmid pBB_CA. Error bars represent standard deviation (n=3).

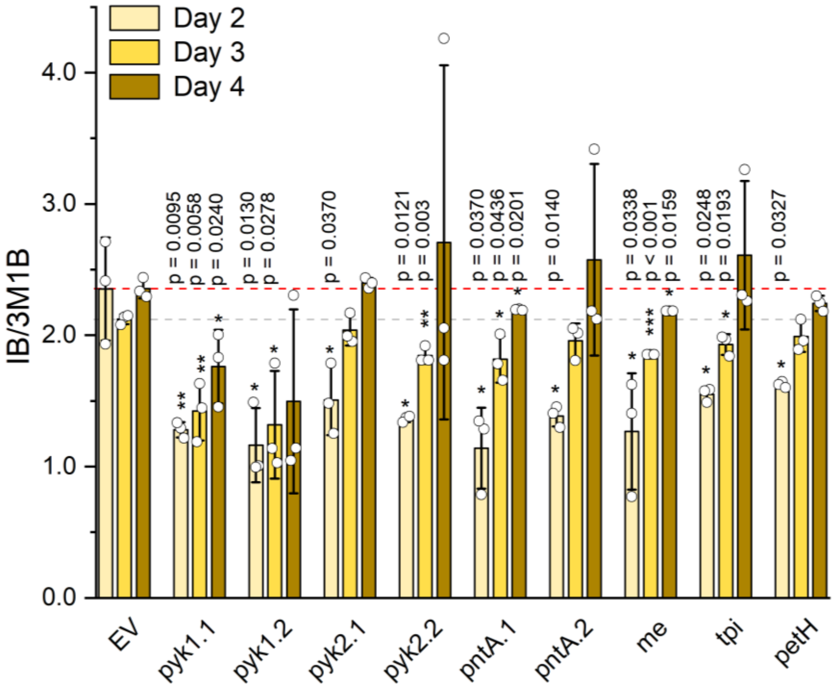

Supplementary Figure 6. IB/3M1B ratio for CRISPRa-targeted strains. EV: negative control – HX11 expressing plasmid pBB_CA. Error bars indicate standard deviation (n=3). p value representation: * < 0.05; ** < 0.01; *** < 0.001.

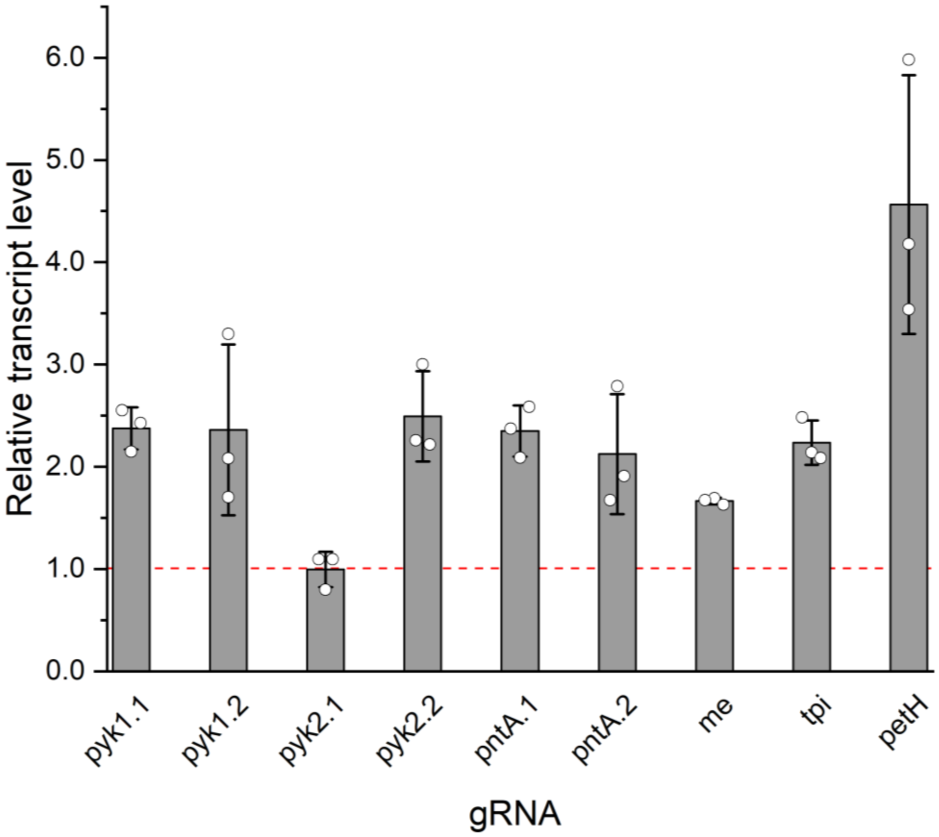

Supplementary Figure 7. Relative transcript levels of candidate genes in response to CRISPRa targeting on day 3. Error bars indicate standard deviation (n=3).

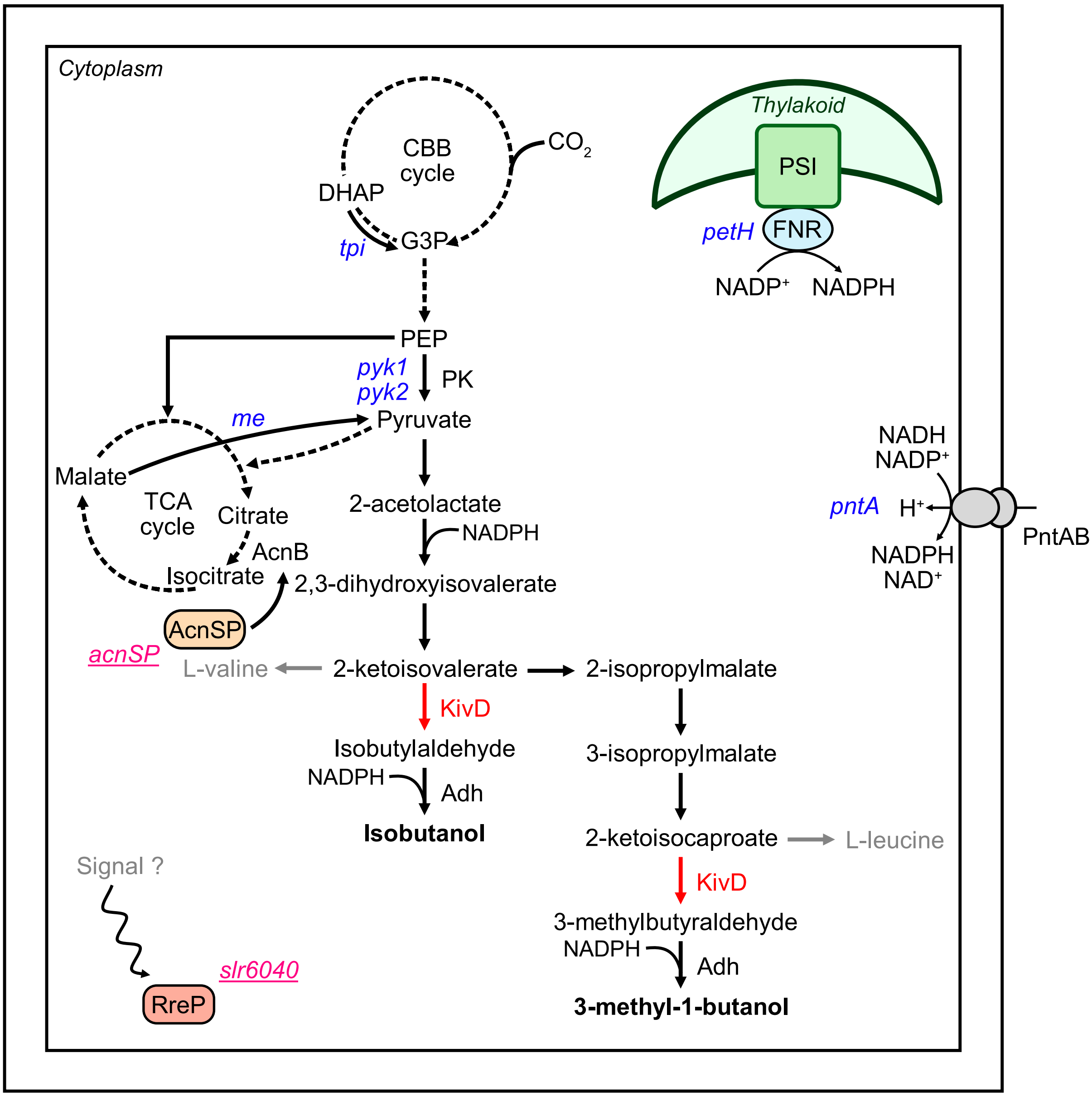

Supplementary Figure 8. Simplified metabolic map for IB and 3M1B biosynthesis. Selected genes were upregulated with CRISPRa (blue) to improve pyruvate availability and NADPH regeneration. The key heterologous enzyme KivD for isobutanol and 3-methyl-1-butanol is highlighted in red. *acnSP* and *slr6040* (pink and underlined) were targeted with the CRISPRa system for downregulation. Abbreviations: AcnB: aconitase; AcnSP: aconitase small protein; Adh: alcohol dehydrogenase; CBB: Calvin-Benson-Bassham cycle; DHAP: dihydroxyacetone phosphate; FNR: ferredoxin-NADP$^+$ oxidoreductase; G3P: glyceraldehyde-3-phosphate; KivD: α-ketoisovalerate decarboxylase; ME: malic enzyme; PEP: phosphoenolpyruvate; PK: pyruvate kinase; PntAB: pyridine nucleotide transhydrogenase; PSI: photosystem I; TCA cycle: tricarboxylic acid cycle.

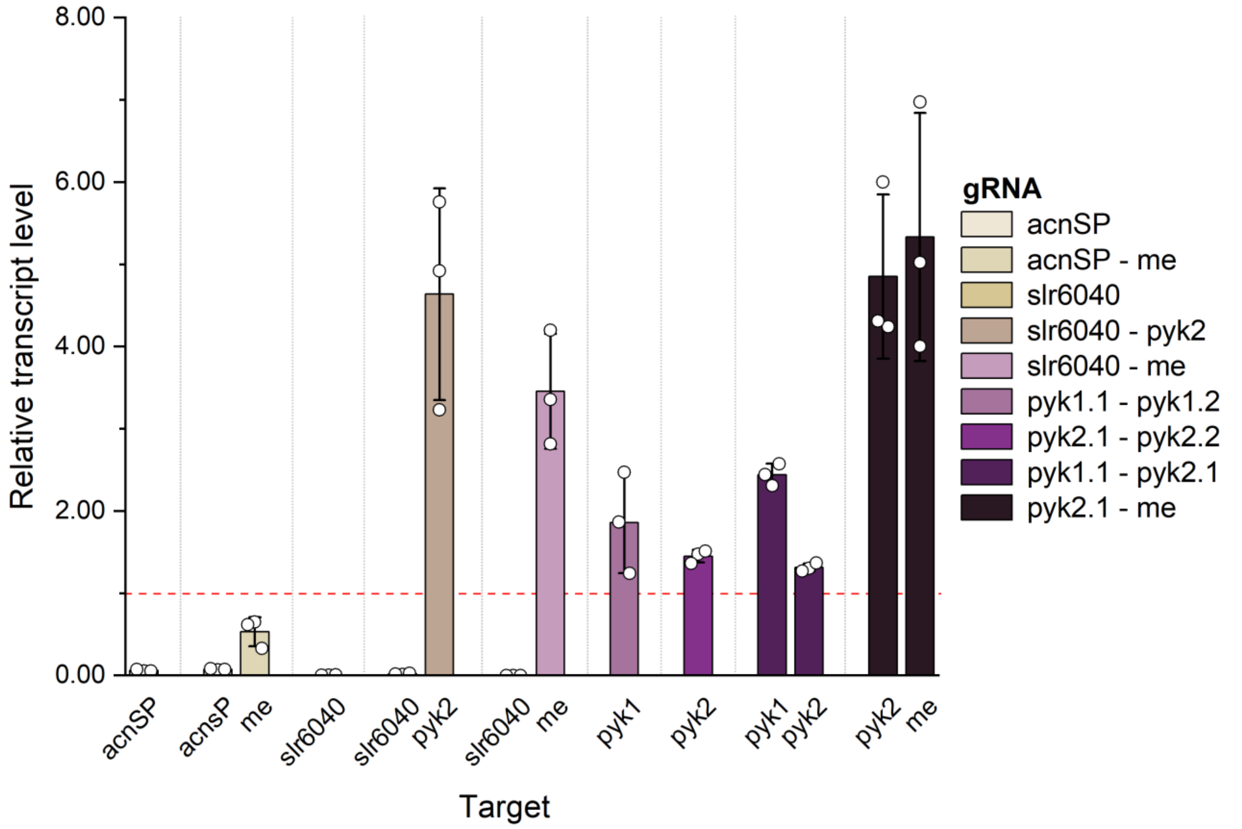

Supplementary Figure 9. Relative transcript levels of multiplexed gene targets under CRISPRa-mediated upregulation and repression on day 2. Error bars indicate standard deviation (n=3).

References

1. Williams, J. G. K. Construction of specific mutations in Photosystem II photosynthetic reaction center by genetic engineering methods in *Synechocystis* 6803. *Methods Enzymol.* **167**, 766–778 (1988).

2. Xie, H. & Lindblad, P. Expressing 2-keto acid pathway enzymes significantly increases photosynthetic isobutanol production. *Microb. Cell Fact.* **21**, 1–17 (2022).

3. Xie, H., Bourgade, B., Stensjö, K. & Lindblad, P. dCas12a-mediated CRISPR interference for multiplex gene repression in cyanobacteria for enhanced isobutanol and 3-methyl-1-butanol production. Preprint available at diva2:1895139 (2024).

4. Xie, H., Kjellström, J. & Lindblad, P. Sustainable production of photosynthetic isobutanol and 3-methyl-1-butanol in the cyanobacterium *Synechocystis* sp. PCC 6803. *Biotechnol. Biofuels Bioprod.* **16**, 1–17 (2023).

5. Behle, A., Saake, P., Germann, A. T., Dienst, D. & Axmann, I. M. Comparative dose-response analysis of inducible promoters in cyanobacteria. *ACS Synth. Biol.* **9**, 843–855 (2020).

6. Knoot, C. J., Biswas, S. & Pakrasi, H. B. Tunable repression of key photosynthetic processes using Cas12a CRISPR interference in the fast-growing cyanobacterium *Synechococcus* sp. UTEX 2973. *ACS Synth. Biol.* **9**, 132–143 (2020).
