# Supplementary material for: Development of a CRISPR activation system for targeted gene upregulation in Synechocystis sp. PCC 6803
Source: Commun Biol. 2025 May 21;8:772. doi: 10.1038/s42003-025-08164-y (PMC12095680; doi:10.1038/s42003-025-08164-y)
Supplement: Supplementary file 2 — Description of Additional Supplementary Materials [file 42003_2025_8164_MOESM2_ESM.pdf]

Description of Additional Supplementary Files

File name: Supplementary Data 1

Description: The main data supporting the findings of this study
