# Supplementary material for: Development of a CRISPR activation system for targeted gene upregulation in Synechocystis sp. PCC 6803
Source: Commun Biol. 2025 May 21;8:772. doi: 10.1038/s42003-025-08164-y (PMC12095680; doi:10.1038/s42003-025-08164-y)
Supplement: Supplementary file 3 — Supplementary Data 1 [file 42003_2025_8164_MOESM3_ESM.pdf]

Supplementary Data 1

List of Figures

Main text
Figure 2. GFP fluorescence in response to CRISPRa targeting
Figure 3. Correlation between promoter strength and activation levels
Figure 4. IB and 3M1B production in response to *kivD* ^{S286T} upregulation
Figure 5. Transcript levels of *kivD* ^{S286T} in response to CRISPRa targeting
Figure 7. CRISPRa-mediated target mapping for IB and 3M1B production
Figure 8. CRISPRa multiplexing for IB and 3M1B biosynthesis

Supplementary Material
Suppl. Figure 1. Tool stability
Suppl. Figure 2. Correlation between inducer concentration and activation levels
Suppl. Figure 3. Growth profiles and IB/3M1B titres of *kivD* ^{S286T}-activated strains
Suppl. Figure 4. Growth profiles of CRISPRa-targeted strains for target mapping
Suppl. Figure 5. IB/3M1B ratio of CRISPRa-targeted strains
Suppl. Figure 6. Transcript levels of CRISPRa-targeted endogenous genes
Suppl. Figure 8. Transcript levels of candidate targets in response to CRISPRa multiplexing

Figure 2b *Fluorescence/OD750*

Uninduced

EV	-48	-97 (NLS)	-108	-144 (NLS)	-156	-251	-328	-108/-156	CDS
337746.22	386628.87	385070.67	422957.67	409132.53	403948.55	333417.74	359820.67	382394.31	386015.99
398414.79	395431.37	394070.66	403583.63	380128.08	411421.05	367938.57	352032.6	402800.95	413169.37
389980.82	401751.09	419044.61	379317.14	390187.68	403851.06	403983.31	423892.71	387478.99	461710.06
391821.85	378043.48		400906.02		397276.06				430798.69
375231.76	402173.77		378217.85		407694.16				432254.41
362989.42	386859.65		365798.83		425257.78				394070.66
	385070.67		386039.43		398183.86				393799.4
	418802.6		391949.43		432476.77				417114.49
	371154.2		419044.61		451561.92				407491.02
	400886.64		390270.56		408841.81				396174.23

Induced

EV	-48	-97 (NLS)	-108	-144 (NLS)	-156	-251	-328	-108/-156	CDS
485796	519519	674571	575495	544929	582727	487452	466256	605257	115944
468891	516054	613983	539587	741132	524710	544557	512170	631725	132230
488114	532518	639865	583562	714800	557448	512841	627828	785114	130354
513534	511767	590922	541792	713744	593286	535090	607507	727257	126120
530919	502090	595398	583856	775890	532719	550278	525737	680913	108939
461436	482740		548873	608788	574881	537867	584929	744607	97539
528853	481742		563306	592236	544317	551726	562017	759659	102314
528906	504881		616714	613469	586195	505669	527928	688255	114614
	494134		599172	749121	524950	631162	519463	833407	105981
	495638		567192	689620	567299	570944		665006	99459

Figure 2c *Fold-activation*

-48	-97 (NLS)	-108	-144 (NLS)	-156	-251	-328	-108/-156	CDS
0.98	1.34	1.09	1.03	1.10	0.92	0.88	1.14	0.22
0.98	1.22	1.02	1.40	0.99	1.03	0.97	1.19	0.25
1.01	1.27	1.10	1.35	1.05	0.97	1.19	1.48	0.25
0.97	1.17	1.02	1.35	1.12	1.01	1.15	1.38	0.24
0.95	1.18	1.10	1.47	1.01	1.04	0.99	1.29	0.21
0.91		1.04	1.15	1.09	1.02	1.11	1.41	0.18
0.91		1.07	1.12	1.03	1.04	1.06	1.44	0.19
0.95		1.17	1.16	1.11	0.96	1.00	1.30	0.22
0.93		1.13	1.42	0.99	1.19	0.98	1.58	0.20
0.94		1.07	1.30	1.07	1.08		1.26	0.19

Figure 3b

J23116		
EV	gRNA	Fold activation
513395	788972	1.41
507030	682900	1.22
602761	618954	1.11
604163	656976	1.18
564777	631750	1.13

J23119		
EV	gRNA	Fold activation
496690	621974	1.22
533998	629963	1.24
514697	525341	1.03
505780	551553	1.08
496705	553790	1.09

J23107		
EV	gRNA	Fold activation
200821	296174	1.55
213755	330844	1.73
224262	344937	1.81
155028	210709	1.10
160814	260284	1.36

J23101		
EV	gRNA	Fold activation
67716	102595	1.50
73977	116975	1.71
60248	177411	2.59
69068	205312	3.00
70974	176008	2.57

Figure 4a *Relative titre*

	Day 4		Day 8	
	IB	3M1B	IB	3M1B
EV	1.09	1.20	1.21	1.57
	1.00	0.94	0.91	0.77
	0.91	0.86	0.86	0.62
ddh	1.27	1.68	1.14	1.06
	0.90	0.88	1.18	1.18
	1.17	1.72	1.27	1.52

Figure 4b *Relative titre*

	Day 4		Day 8	
	IB	3M1B	IB	3M1B
EV	1.03	1.02	1.16	1.26
	0.98	0.97	0.94	0.88
	0.99	1.01	0.90	0.86
ddh	0.84	0.66	0.89	0.81
	0.82	0.70	0.83	0.63
	0.96	0.95	1.06	1.16
NS1	1.26	1.53	1.07	1.12
	0.96	1.00	1.07	1.15
	1.10	1.44	1.07	1.24
NS1-ddh	1.35	1.67	1.09	1.20
	1.33	1.73	1.07	1.16
	1.01	1.03	0.87	0.70

Figure 4c *Relative titre*

	Day 4		Day 8	
	IB	3M1B	IB	3M1B
EV	1.00	0.96	1.06	0.98
	1.01	1.01	1.00	1.02
	0.99	1.03	0.94	1.00
ddh	0.70	0.30	0.94	0.92
	0.97	1.06	0.90	0.73
	0.71	0.32	0.98	1.04
NS1	0.64	0.23	0.93	0.92
	0.91	1.00	0.78	0.61
	0.91	0.88	0.82	0.68
sll1564	1.05	1.17	1.00	1.06
	1.30	1.79	1.22	1.49
	1.11	1.26	1.11	1.31
NS1-ddh	0.81	0.57	0.80	0.44
	0.96	0.85	1.02	0.87
	0.87	0.75	1.03	1.05
NS1-sll1564	1.05	1.18	1.01	1.17
	1.12	1.23	1.09	1.27
	1.19	1.39	1.04	1.17
ddh-sll1564	1.21	1.45	1.14	1.39
	0.83	0.67	0.90	0.87
	1.06	1.24	1.06	1.29
Triple	1.08	1.16	1.11	1.36
	1.18	1.42	1.01	1.15
	1.12	1.37	1.00	1.17

Figure 5 *Relative expression*

ddh_kivD	
EV	ddh
1.04	2.86
0.96	3.32
1.00	3.40

	HX11			
	EV	ddh	NS1	NS1-ddh
Flag	0.97	1.07	1.11	0.80
	0.96	1.10	1.08	0.87
	1.08	1.04	1.04	0.82
His	0.89	1.13	1.34	1.13
	0.73	1.09	1.30	1.14
	1.06	1.05	1.30	1.35

	HX51							
	EV	ddh	NS1	sll1564	NS1-ddh	NS1-sll1564	ddh-sll1564	Triple
Flag	1.15	0.76	1.33	0.80	2.75	1.45	0.96	1.96
	0.91	1.29	0.40	0.71	2.44	1.58	1.06	1.71
	0.96	1.28	1.03	0.79	1.07	1.43	0.97	1.93
His	1.25	0.80	1.17	0.67	2.65	1.78	1.15	2.10
	0.76	1.10	0.42	0.87	2.45	1.68	1.12	1.48
	1.06	1.13	0.89	1.25	2.54	1.62	1.02	1.69
Total	1.04	0.30	0.53	0.97	1.69	0.58	0.46	0.98
	1.04	0.40	0.45	0.98	1.92	0.75	0.60	1.02
	0.93	0.30	0.53	1.81	1.83	0.68	0.57	1.21

Figure 7a *IB titres*

	Day 2	Day 3	Day 4
EV	2.76<br>2.96<br>3.08	2.56<br>2.60<br>3.40	3.76<br>3.59<br>3.61
pyk1.1	5.69<br>6.11<br>5.05	8.71<br>9.23<br>5.85	8.58<br>10.61<br>7.49
pyk1.2	5.95<br>7.05<br>5.04	13.32<br>14.96<br>5.22	15.64<br>14.25<br>4.97
pyk2.1	4.22<br>4.00<br>4.11	4.37<br>3.60<br>3.93	4.38<br>4.46<br>4.42
pyk2.2	3.63<br>4.14<br>3.64	4.19<br>4.99<br>3.81	4.62<br>2.54<br>4.62
pntA.1	4.90<br>5.36<br>3.92	5.14<br>4.25<br>3.37	5.24<br>4.82<br>4.41
pntA.2	3.66<br>3.95<br>4.45	3.58<br>4.29<br>5.10	3.43<br>4.93<br>5.34
ME	4.69<br>5.05<br>5.04	4.01<br>4.01<br>4.01	5.34<br>5.01<br>4.67
tpi	4.08<br>3.14<br>4.60	4.32<br>3.65<br>4.34	5.53<br>3.35<br>4.82
petH	4.27<br>4.13<br>3.98	4.23<br>5.18<br>4.43	5.07<br>5.36<br>4.48

Figure 7b *3M1B titres*

	Day 2	Day 3	Day 4
EV	1.02	1.20	1.54
	1.53	1.25	1.54
	1.28	1.58	1.58
pyk1.1	4.40	6.03	4.69
	4.58	7.77	7.30
	4.15	3.58	3.74
pyk1.2	5.92	12.95	13.71
	7.09	13.14	13.62
	3.38	2.92	2.16
pyk2.1	2.36	2.24	1.86
	3.19	1.80	1.83
	2.77	1.81	1.84
pyk2.2	2.65	2.18	1.09
	3.10	2.76	1.40
	2.64	2.10	2.25
pntA.1	3.81	3.10	2.38
	6.81	2.39	2.20
	2.91	1.68	2.01
pntA.2	2.51	1.78	1.00
	2.81	2.09	2.32
	3.43	2.82	2.44
ME	3.33	2.17	2.44
	6.55	2.17	2.29
	3.10	2.17	2.14
tpi	2.74	2.20	2.45
	1.98	1.84	1.03
	2.92	2.36	2.09
petH	2.59	2.16	2.25
	2.54	2.74	2.33
	2.48	2.09	2.05

Figure 7c *Fold-increase*

	IB	3M1B
pyk1.1	2.06	4.32
	2.06	2.99
	1.64	3.25
pyk1.2	2.15	5.81
	2.38	4.63
	1.64	2.65
pyk2.1	1.53	2.31
	1.35	2.08
	1.33	2.18
pyk2.2	1.32	2.60
	1.40	2.02
	1.18	2.07
pntA.1	1.77	3.74
	1.81	4.44
	1.27	2.28
pntA.2	1.32	2.47
	1.33	1.83
	1.45	2.69
ME	1.70	3.27
	1.71	4.28
	1.64	2.43
tpi	1.48	2.69
	1.06	1.29
	1.50	2.29
petH	1.55	2.55
	1.39	1.66
	1.29	1.95

Figure 7d *IB/3M1B ratio*

	Day 2	Day 3	Day 4
EV	2.71	2.14	2.44
	1.93	2.08	2.33
	2.41	2.15	2.29
pyk1.1	1.29	1.45	1.83
	1.33	1.19	1.45
	1.22	1.63	2.00
pyk1.2	1.00	1.03	1.14
	0.99	1.14	1.05
	1.49	1.79	2.30

Figure 8a *2 gRNAs targeting 1 single gene*

	IB	3M1B
pyk1.1	6.34<br>7.40	3.39<br>3.86
pyk1.2	8.83<br>7.71	3.97<br>4.59
pyk1.1 - pyk1.2	4.65<br>7.24<br>6.82	4.71<br>4.75<br>5.76
pyk2.1	3.10<br>4.47	1.65<br>2.68
pyk2.2	5.82<br>5.40	3.57<br>3.08
pyk2.1 - pyk2.2	4.77<br>4.00<br>4.36	3.68<br>3.39<br>2.88

Figure 8b *Simultaneous targeting of 2 genes*

	IB	3M1B
pyk1.1	6.34<br>7.40	3.39<br>3.86
pyk1.1 - pyk2.1	20.42<br>12.07<br>14.35	14.35<br>13.71<br>12.04
pyk2.1	3.10<br>4.47	1.65<br>2.68
pyk2.1 - me	17.62<br>21.06<br>14.59	12.87<br>10.97<br>10.38
me	7.17<br>6.60	5.05<br>4.66

Figure 8c *CRISPRi*

	IB	3M1B
EV	6.02	4.85
	4.56	3.98
	4.08	4.44
slr6040	6.70	4.68
	7.04	7.93
	7.20	5.51
slr6040 - pyk2.1	5.88	5.53
	8.30	6.82
	6.56	4.10
slr6040 - me	6.13	8.06
	4.83	6.29
	3.53	4.51
acnSP	5.56	7.39
	8.01	4.43
	5.40	7.56
acnSP - me	15.91	13.96
	19.09	12.51
	17.09	16.03

Figure 8d *Fold-change*

Multiplexed gRNA	Single target	IB	3M1B
pyk1.1 - pyk2.1	pyk1.1	2.97	3.96
		1.76	3.78
		2.09	3.32
	pyk2.1	5.40	6.63
		3.19	6.33
		3.79	5.56
pyk2.1 - ME	pyk2.1	4.66	5.94
		5.57	5.06
		3.86	4.79
	me	2.56	2.65
		3.06	2.26
		2.12	2.14
acnSP - me	acnSP	2.52	2.16
		3.02	1.94
		2.70	2.48
	me	2.31	2.88
		2.77	2.58
		2.48	3.30
slr6040 - pyk2.1	slr6040	0.84	0.92
		1.19	1.13
		0.94	0.68
	pyk2.1	1.56	2.55
		2.19	3.15
		1.74	1.89
slr6040 - me	slr6040	0.88	1.33
		0.69	1.04
		0.51	0.75
	me	0.89	1.66
		0.70	1.29
		0.51	0.93

Suppl. Figure 2a *GFP fluorescence*

	24	48	72	96
EV	591194<br>634036<br>602043	527410<br>497581<br>522846	426377<br>420783<br>448392	435274<br>456945<br>459926
-48	583400<br>559912<br>570708	509460<br>513667<br>522373	446856<br>459288<br>462425	494389<br>452203<br>491443
-108	751538<br>827289<br>1459000	618048<br>606282<br>708119	527986<br>492738<br>520500	565775<br>592701<br>520009
-156	780320<br>924333<br>651514	596016<br>600831<br>466636	485276<br>540839<br>459393	545234<br>597210<br>589659
CDS	286548<br>283590<br>256220	198873<br>219225<br>177966	196028<br>197959<br>195197	152794<br>139127<br>139344

Suppl. Figure 2b *Fold-activation*

	48	96
-48	0.80<br>0.89<br>1.23	0.88<br>0.92<br>0.87
-108	0.83<br>0.96<br>1.13	1.00<br>1.05<br>0.98
-156	0.91<br>1.19<br>1.38	1.37<br>1.25<br>1.27
CDS	0.07<br>0.08<br>0.10	2.49E-04<br>2.50E-04<br>2.43E-04

Suppl. Figure 3 *GFP fluorescence in response to different rhamnose concentrations*

	3 mM	6 mM	9 mM
EV	485796	436752	481713
	489146	381677	429714
	437409	448818	471546
-97 NLS	674571	649152	619417
	613983	622800	680799
	639865	617415	742180
-108	575495	577868	601519
	539587	586197	601872
	519676	538727	633037
-144 NLS	529818	526806	536004
	610806	492067	550125
	544929	487799	613125
-156	587678	495740	588525
	505344	443532	619165
	531584	587135	536507

Suppl. Figure 4a *ddh_kivD strains*

Growth

			Strain	
			EV	ddh
Day	0	Mean	0.43	0.10
		SD	0.00	0.00
	1	Mean	0.43	0.35
		SD	0.12	0.11
	2	Mean	1.59	1.50
		SD	0.41	0.71
	3	Mean	2.81	1.96
		SD	0.33	1.04
	4	Mean	3.81	2.82
		SD	0.20	0.95
	5	Mean	4.37	3.49
		SD	0.37	0.72
	6	Mean	4.17	3.49
		SD	0.24	0.81
	7	Mean	3.77	4.25
		SD	0.36	0.53
	8	Mean	3.39	3.24
		SD	0.29	1.31
	9	Mean	3.34	3.46
		SD	0.40	0.60
	10	Mean	2.42	3.19
		SD	0.17	0.59

Product formation

		Strain			
		EV		ddh	
		IB	3M1B	IB	3M1B
Day	3	2.98	1.09	2.76	1.39
		2.42	0.81	2.08	0.88
		2.08	0.79	2.38	0.69
	4	3.12	1.25	3.63	1.60
		2.86	1.06	2.57	1.11
		2.61	1.08	3.35	1.75
	6	5.01	2.34	3.23	1.28
		4.02	1.73	4.93	2.05
		3.70	1.50	5.01	2.53
	8	6.50	3.58	6.11	2.44
		4.86	2.28	6.29	2.66
		4.58	2.01	6.79	3.29
	10	7.92	4.27	6.75	2.81
		5.79	2.85	6.25	2.67
		5.44	2.51	6.85	2.86

Suppl. Figure 4b *HX11 strains*

Growth

			Strain			
			EV	ddh	NS1	NS1-ddh
Day	0	Mean	0.10	0.10	0.10	0.10
		SD	0.00	0.00	0.00	0.00
	1	Mean	0.26	0.22	0.23	0.22
		SD	0.01	0.01	0.02	0.02
	2	Mean	0.40	0.32	0.35	0.30
		SD	0.02	0.01	0.03	0.02
	3	Mean	1.57	1.28	1.58	0.92
		SD	0.21	0.18	0.28	0.12
	4	Mean	3.25	3.31	3.25	2.24
		SD	0.13	0.31	0.44	0.15
	5	Mean	4.64	4.29	3.90	3.48
		SD	0.18	0.40	0.43	0.23
	6	Mean	4.62	4.58	4.75	4.61
		SD	0.19	0.19	0.13	0.29
	7	Mean	4.17	3.79	4.78	4.45
		SD	0.20	1.07	0.38	0.82
	8	Mean	4.12	4.12	4.78	5.07
		SD	0.54	0.48	0.55	0.17
	9	Mean	3.39	3.30	3.89	4.21
		SD	0.67	0.66	0.22	0.32
	10	Mean	2.91	3.45	3.73	3.98
		SD	0.26	0.48	0.30	0.25

Suppl. Figure 4b *HX11 strains*

Product formation

		Strain							
		EV		ddh		NS1		NS1-ddh	
		IB	3M1B	IB	3M1B	IB	3M1B	IB	3M1B
Day	3	2.90	1.36	3.16	1.51	4.38	2.22	4.79	2.50
		2.94	1.41	3.00	1.43	3.32	1.57	5.40	3.04
		3.85	1.79	3.93	2.03	3.19	1.57	3.48	1.81
	4	4.00	1.64	3.25	1.36	4.89	2.10	5.24	2.18
		3.82	1.64	3.21	1.45	3.75	1.72	5.19	2.31
		3.85	1.68	3.72	1.63	4.26	2.23	3.92	1.68
	6	8.37	3.45	7.88	3.45	8.82	3.73	9.73	4.18
		8.18	3.68	7.92	3.55	8.27	3.56	9.05	3.95
		8.12	3.77	7.92	3.22	9.58	4.53	7.70	3.25
	8	13.98	5.98	10.67	5.00	12.82	5.76	13.13	6.03
		11.24	5.16	9.99	4.35	12.81	5.90	12.91	5.94
		10.82	5.21	12.79	5.99	12.85	6.40	10.40	4.52
	10	18.40	8.59	12.21	5.80	18.53	8.81	13.71	6.45
		12.72	5.98	14.51	6.61	15.13	6.87	16.95	8.35
		11.40	5.76	12.86	6.11	16.07	7.92	14.43	7.17

Suppl. Figure 4c *HX51 strains*

Growth

			Strain							
			EV	ddh	NS1	sll1564	NS1-ddh	NS1-sll1564	ddh-sll1564	Triple
Day	0	Mean	0.10	0.10	0.10	0.10	0.10	0.10	0.10	0.10
		SD	0.00	0.00	0.00	0.00	0.00	0.00	0.00	0.00
	1	Mean	0.34	0.37	0.24	0.25	0.29	0.33	0.26	0.32
		SD	0.01	0.02	0.02	0.04	0.05	0.04	0.05	0.04
	2	Mean	1.45	1.63	1.48	1.39	1.34	1.47	1.41	1.40
		SD	0.12	0.19	0.10	0.25	0.12	0.10	0.15	0.14
	3	Mean	2.68	3.11	3.13	3.06	2.67	3.02	3.07	3.14
		SD	0.29	0.30	0.21	0.29	0.14	0.17	0.12	0.30
	4	Mean	3.46	3.46	3.55	3.51	3.80	3.60	3.61	3.44
		SD	0.15	0.10	0.12	0.13	0.12	0.11	0.28	0.14
	5	Mean	3.60	3.00	3.10	3.18	3.93	3.12	3.12	3.39
		SD	0.35	0.42	0.27	0.29	0.29	0.26	0.27	0.38
	6	Mean	3.94	3.15	3.52	3.68	4.38	3.72	3.52	3.54
		SD	0.23	0.55	0.43	0.43	0.12	0.10	0.32	0.29
	7	Mean	3.86	2.76	3.00	3.12	3.69	3.47	3.14	3.31
		SD	0.60	0.83	0.59	0.37	0.34	0.15	0.36	1.10
	8	Mean	3.14	2.62	2.73	2.89	3.46	3.23	2.79	3.01
		SD	0.24	0.74	0.70	0.37	0.25	0.09	0.24	0.31
	9	Mean	2.49	2.50	2.40	2.22	2.35	2.53	2.08	2.24
		SD	0.37	0.16	0.33	0.29	0.35	0.25	0.38	0.38
	10	Mean	2.38	2.27	2.24	2.01	2.50	2.33	2.09	2.18
		SD	0.31	0.20	0.20	0.24	0.04	0.27	0.13	0.22

Suppl. Figure 4c *HX51 strains*

Product formation

		Strain											
		EV		ddh		NS1		sll1564		NS-ddh		NS1-sll1564	
		IB	3M1B	IB	3M1B	IB	3M1B	IB	3M1B	IB	3M1B	IB	3M1B
Day	3	4.83	1.94	5.28	2.08	5.03	2.11	4.73	2.12	4.49	1.62	5.24	2.15
		5.77	2.18	4.64	2.10	4.07	1.81	5.80	2.26	5.57	2.17	5.98	2.36
		5.03	1.98	5.37	2.14	4.32	1.82	5.60	2.26	4.88	1.95	6.00	2.48
	4	6.31	2.77	4.40	1.73	4.03	1.70	6.63	3.24	5.11	2.20	6.62	3.27
		6.36	2.89	6.11	3.17	5.72	3.14	8.17	4.31	6.02	2.59	7.04	3.22
		6.22	3.02	4.44	1.77	5.75	2.79	6.98	3.34	5.47	2.55	7.47	3.50
	6	10.90	4.89	9.56	4.85	9.28	5.25	9.44	4.97	8.06	3.56	9.72	5.59
		10.42	5.05	8.68	4.29	7.37	4.02	13.05	6.93	11.69	5.10	11.15	5.75
		9.73	5.25	9.88	5.13	9.56	5.18	10.61	5.60	10.90	5.20	10.89	5.72
	8	12.98	5.88	11.49	6.25	11.42	6.28	12.25	6.75	9.80	4.07	12.38	7.39
		12.26	6.48	11.00	5.30	9.61	5.22	14.98	8.08	12.46	5.40	13.37	7.52
		11.54	6.70	12.01	6.76	10.05	5.47	13.64	7.59	12.60	6.50	12.77	7.18
	10	12.72	7.20	14.36	8.10	12.59	7.51	19.44	10.36	17.25	9.75	14.75	8.93
		13.59	8.00	12.44	7.27	14.20	8.46	14.79	8.39	14.55	8.22	13.42	7.85
		12.77	7.23	14.94	8.43	12.37	7.38	17.51	9.33	*	*	18.02	9.98

* : chlorotic (dead) culture

Suppl. Figure 4c *HX51 strains*

Product formation

		Strain			
		ddh-sll1564		Triple	
		IB	3M1B	IB	3M1B
Day	3	6.04	2.54	5.77	2.21
		4.60	1.86	5.91	2.51
		5.27	2.22	5.37	2.34
	4	7.60	3.58	6.80	3.13
		5.25	2.42	7.42	3.58
		6.69	3.42	7.06	3.60
	6	11.44	6.28	11.47	6.01
		8.90	4.77	10.90	6.08
		10.42	6.15	10.04	5.90
	8	13.92	7.94	13.57	7.69
		10.99	6.17	12.43	7.24
		13.05	7.77	12.30	7.41
	10	16.95	9.64	16.24	9.53
		12.24	7.06	13.61	8.11
		16.19	9.20	15.78	9.67

Suppl. Figure 5 *Growth profiles*

	Day 0		Day 1		Day 2		Day 3		Day 4	
	Mean	SD	Mean	SD	Mean	SD	Mean	SD	Mean	SD
EV	0.1	0	0.27	0.04	0.40	0.02	1.57	0.21	3.25	0.13
pyk1.1	0.1	0	0.13	0.01	0.24	0.05	0.49	0.24	1.09	0.43
pyk1.2	0.1	0	0.14	0.02	0.25	0.14	0.48	0.49	1.06	1.25
pyk2.1	0.1	0	0.21	0.03	0.41	0.10	1.08	0.16	2.98	0.17
pyk2.2	0.1	0	0.19	0.02	0.40	0.05	1.23	0.15	2.67	0.36
pntA.1	0.1	0	0.14	0.01	0.31	0.06	1.09	0.34	2.70	0.50
pntA.2	0.1	0	0.18	0.00	0.43	0.06	1.48	0.34	2.80	0.20
me	0.1	0	0.19	0.01	0.36	0.01	1.24	0.11	2.59	0.06
tpi	0.1	0	0.20	0.02	0.48	0.06	1.27	0.13	2.57	0.10
petH	0.1	0	0.20	0.03	0.46	0.09	1.29	0.23	2.93	0.45

Suppl. Figure 6 *IB/3M1B ratio*

	Day 2	Day 3	Day 4
EV	2.71	2.14	2.44
	1.93	2.08	2.33
	2.41	2.15	2.29
pyk1.1	1.29	1.45	1.83
	1.33	1.19	1.45
	1.22	1.63	2.00
pyk1.2	1.00	1.03	1.14
	0.99	1.14	1.05
	1.49	1.79	2.30
pyk2.1	1.79	1.95	2.36
	1.25	1.99	2.44
	1.48	2.17	2.40
pyk2.2	1.37	1.92	4.26
	1.34	1.81	1.81
	1.38	1.81	2.06
pntA.1	1.29	1.66	2.20
	0.79	1.78	2.20
	1.35	2.01	2.19
pntA.2	1.45	2.02	3.42
	1.41	2.05	2.12
	1.30	1.81	2.18
ME	1.41	1.85	2.18
	0.77	1.85	2.18
	1.62	1.85	2.18
tpi	1.49	1.97	2.26
	1.59	1.99	3.26
	1.58	1.84	2.31
petH	1.65	1.96	2.25
	1.62	1.89	2.30
	1.60	2.12	2.18

Suppl. Figure 7 *Relative transcript levels*

pyk1.1	2.15<br>2.55<br>2.43
pyk1.2	3.30<br>2.08<br>1.70
pyk2.1	1.10<br>0.80<br>1.10
pyk2.2	2.22<br>2.26<br>3.00
pntA.1	2.09<br>2.37<br>2.59
pntA.2	1.91<br>1.67<br>2.79
me	1.69<br>1.67<br>1.63
tpi	2.14<br>2.48<br>2.08
petH	3.54<br>4.18<br>5.98

Suppl. Figure 9 *Relative transcript levels*

gRNA	Single target	
acnSP	*acnSP*	0.06<br>0.07<br>0.05
acnSP - me	*acnSP*	0.07<br>0.08<br>0.07
	me	0.65<br>0.62<br>0.33
slr6040	*slr6040*	0.00<br>0.00<br>0.01
slr6040 - pyk2.1	*slr6040*	0.01<br>0.02<br>0.03
	pyk2	4.92<br>3.23<br>5.76
slr6040 - me	*slr6040*	0.00<br>0.00<br>0.00
	me	2.82<br>4.20<br>3.36

gRNA	Single target	
pyk1.1 - pyk1.2	*pyk1*	2.47<br>1.24<br>1.87
pyk2.1 - pyk2.2	*pyk2*	1.47<br>1.36<br>1.51
pyk1.1 - pyk2.1	*pyk1*	2.31<br>2.44<br>2.57
	pyk2	1.30<br>1.27<br>1.37
pyk2.1 - ME	*pyk2*	6.00<br>4.24<br>4.31
	me	4.00<br>6.97<br>5.02
